# Surgical management and ultrastructural study of choroidal neovascularization in punctate inner choroidopathy after bevacizumab

**DOI:** 10.1007/s12348-011-0050-x

**Published:** 2011-11-27

**Authors:** Sophia I. Pachydaki, Frederick A. Jakobiec, Pooja Bhat, Lucia Sobrin, Norman A. Michaud, Surya V. Seshan, Donald J. D’Amico

**Affiliations:** 1 Department of Ophthalmology, Weill Cornell Medical College, New York, NY USA; 2 David G. Cogan Laboratory of Ophthalmic Pathology, Massachusetts Eye and Ear Infirmary, Harvard Medical School, 243 Charles Street, Boston, MA 02114 USA; 3 Retina Service, Massachusetts Eye & Ear Infirmary, Harvard Medical School, 243 Charles Street, Boston, MA 02114 USA

**Keywords:** Bevacizumab, Choroidal neovascularization, Electron microscopy, Light microscopy, Punctate inner choroidopathy, Submacular surgery

## Abstract

**Purpose:**

This study aims to describe surgical management results and the pathologic features of choroidal neovascularization (CNV) secondary to punctate inner choroidopathy (PIC) following anti-vascular endothelial growth factor treatment.

**Design:**

This study is a case report on the surgical management and ultrastructural study of choroidal neovascularization.

**Methods:**

Clinicopathologic and ultrastructural report of CNV membranes excised from both eyes of one patient was presented.

**Results:**

The right eye responded to bevacizumab, and recurrence 17 months later did not; the left eye never responded. Excision of the active CNVs was performed 3 months after the last injection. In the right eye, there was no recurrence 23 months after surgery. In the left eye, CNV recurred after 6 months, with no response to bevacizumab. Electron microscopy revealed subretinal neovascular tissue and, additionally, Bruch's membrane and inner choroid in the left. In the right eye, lumens of many neovascular channels were occluded by microfibrils and pericytes were infrequent. In the left eye, patent CNV units with pericytes were present. There were scattered macrophages but no lymphocytes in either membrane. An inner focal choroidal lymphocytic infiltrate was discovered.

**Conclusions:**

Submacular surgery did not cause complications following treatment with bevacizumab. Mostly nonfunctional capillaries in the right membrane failed to display pericytes. The left membrane, which was unresponsive to bevacizumab, displayed well-formed neovascular units consistently exhibiting pericytes. A focus of inner choroidal lymphocytic infiltration was found in the left eye despite the absence of overt clinical intraocular inflammation. This is the first pathological study employing human tissue that points to pericytes as a potential critical therapeutic target with the aggravating influence of inner choroidal chronic inflammation in PIC.

## Introduction

Punctate inner choroidopathy (PIC) is an idiopathic disorder representing one point on a spectrum of the multifocal choroiditides. The anterior segment and vitreous are typically free of inflammation. The visual outcome is good unless complicated by submacular choroidal neovascularization (CNV), which may occur bilaterally. Treatment options of CNV in PIC include argon laser photocoagulation, local or systemic corticosteroids or other immunosuppressant agents, intravitreal anti-VEGF agents,photodynamic therapy (PDT), and submacular surgery. In CNV cases with subfoveal involvement, argon laser photocoagulation has been associated with poor visual outcome. Before the introduction of anti-vascular endothelial growth factor (VEGF) agents, surgical excision was the therapeutic option of last resort and only employed after conventional management with corticosteroids or PDT had failed.

Various angiogenic factors have been implicated in the pathogenesis of CNV, with VEGF putatively the key player. The advent of targeted pharmacologic inhibition of VEGF has been a major advance in the management of CNV secondary to age-related macular degeneration (AMD) [1]. There are no randomized clinical trials providing evidence for the efficacy of intravitreal anti-VEGF agents in the treatment of CNV in conditions other than AMD. Nevertheless, given their excellent safety profile, these agents are widely used in all subfoveal CNV cases regardless of etiology, including PIC [2, 3]. It remains unclear why CNV responds in different ways to anti-VEGF treatment. A multifactorial etiology of ocular angiogenesis, including inflammation and mediators other than VEGF, as well as the different levels of vascular maturation of the neovascular complex, has been proposed.

To date, there is no report of the surgical management and ultrastructural study of CNV secondary to choroiditis following intravitreal anti-VEGF treatment. We report herein on the clinical outcome of bilateral surgical excisions of CNV in a PIC case which failed anti-VEGF treatment. Electron microscopic evaluation was performed on the excised specimens in an attempt to elucidate the pathogenesis of the condition and the possible mechanisms of treatment failure.

Informed consent for the research was obtained from the patient. The study is in accordance with HIPAA regulations.

## Clinical findings

**Fig. 1:**
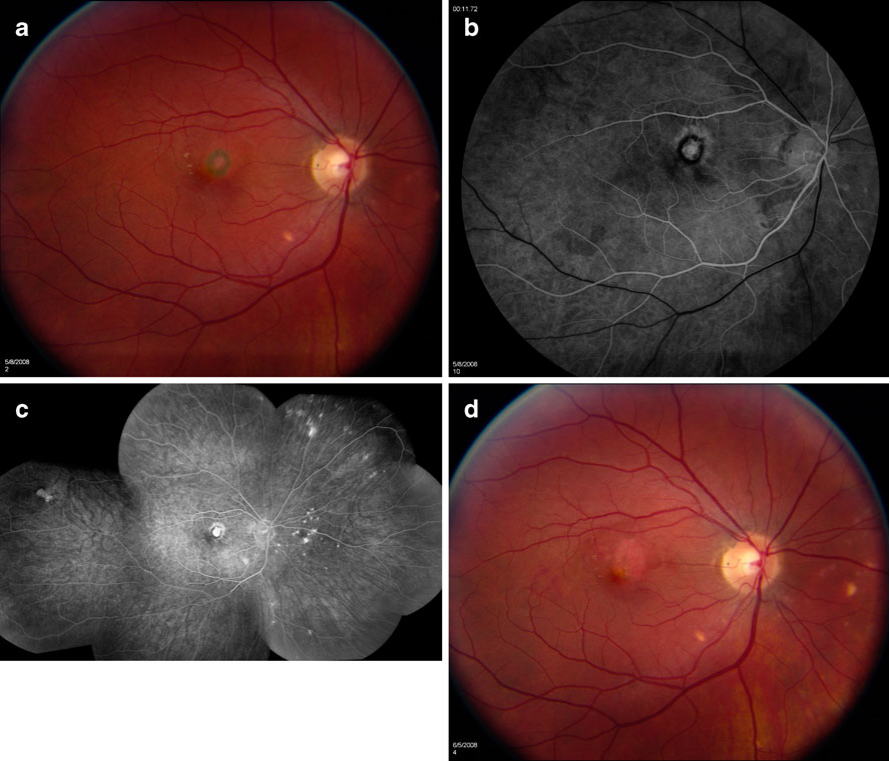
Right eye. **a** Preoperative color photograph demonstrates multiple, small, yellowish punctate lesions at the level of the choroid. A subfoveal CNV membrane with hyperpigmented borders is seen. **b** Early transit of the fluorescein angiogram shows early hyperfluorescence of the CNV lesion. **c** Late phase of the angiogram shows leakage from the CNV along with multiple punctate hyperfluorescent lesions in the peripapillary area and the periphery. **d** Fundus photograph 1 month after surgical excision of CNV showing RPE atrophy

**Fig. 2:**
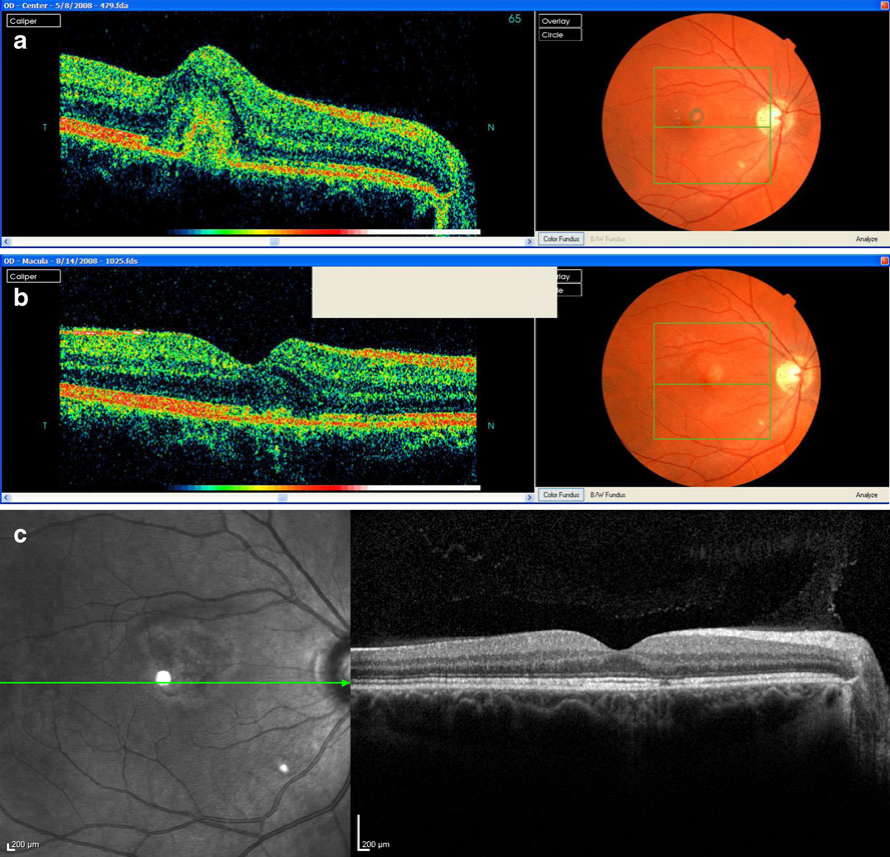
Right eye. **a** (*Top*) OCT before surgical excision of CNV showing hyperreflective area at the level of the RPE corresponding to the CNV. **b** (*Middle*) OCT 3 months after surgical excision of CNV. **c** (*Bottom*) OCT 15 months after surgical excision of CNV

A 42-year-old white woman presented with metamorphopsia in the right eye. Past ocular, medical and family history was unremarkable. Visual acuity was 20/125 in the right and 20/20 in the left. The patient was diagnosed with CNV associated with PIC. The CNV regressed following one intravitreal bevacizumab injection with an improvement in vision to 20/25. It recurred 17 months later with a decrease in vision to 20/40, and a second injection was administered with no response. The patient deferred further intraocular injections and could not tolerate systemic immunosuppression. She presented to us for submacular surgery evaluation. Visual acuity was 20/40 in the right eye and 20/20 in the left. Anterior segment and vitreous examination showed no inflammation. Dilated fundus examination revealed small punctate yellowish chorioretinal lesions bilaterally consistent with choroiditis (Fig. 1a). In the right eye, fluorescein angiography disclosed a classic subretinal neovascular membrane (Fig. 1b, c). Optical coherence tomography (OCT) revealed that the lesion was associated with adjacent intraretinal cystic spaces (Fig. 2a, top).

Surgical removal of the CNV 3 months after the second injection led to improvement in vision to 20/20-1 with resolution of metamorphopsia (Figs. 1d and 2b middle and c bottom). There has been no recurrence at 23 months of follow-up after surgery.

**Fig. 3:**
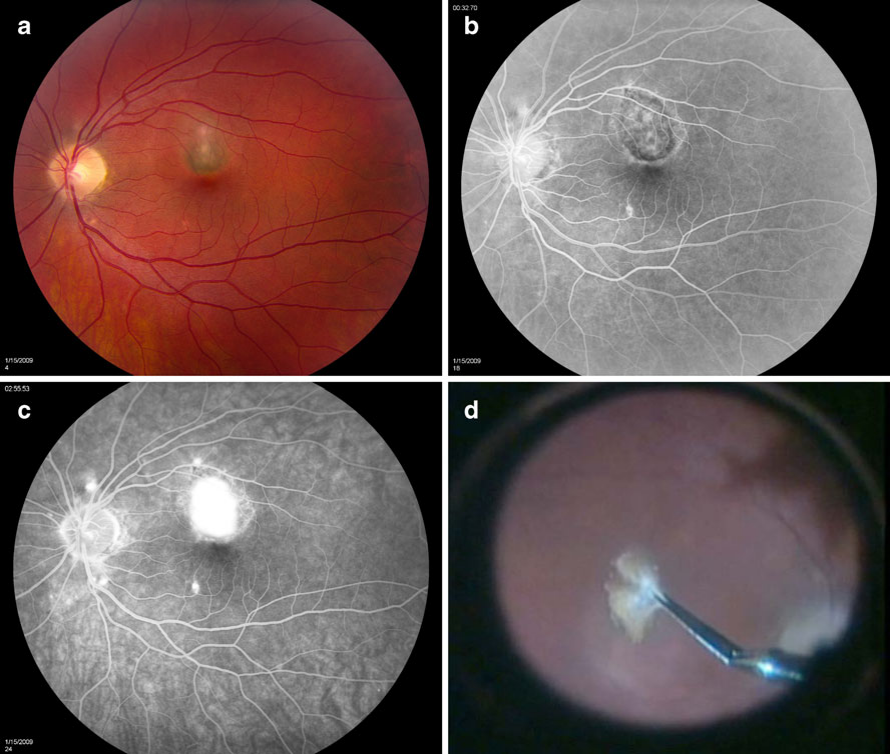
Left eye. **a** Color fundus photograph 3 months after second injection of bevacizumab shows a grayish subfoveal CNV membrane. **b** Early phase of the fluorescein angiogram reveals hyperfluorescence corresponding to the CNV lesion with hypofluorescent borders corresponding to the pigmented borders of the lesion. **c** Late phase of the angiogram reveals leakage of the CNV lesion. **d** Intraoperative view of the excised CNV lesion

CNV developed in the left eye 2 years after failed pharmacotherapy in the right eye. Vision was 20/20-2 with severe symptoms of metamorphopsia. There was no response to two injections of bevacizumab (Fig. 3a–c). Surgical removal of CNV 3 months after the second injection (Fig. 3d) led to resolution of metamorphopsia and improvement of vision to 20/20 at a 3-month follow-up. Six months following surgery, visual acuity decreased to 20/63-2 secondary to recurrence. There was no response to additional treatment with bevacizumab. Patient elected for repeat submacular surgery. Vision improved to 20/32 5 months postoperatively. Ultrastructural studies of that specimen were not performed. Informed consent was obtained from the patient regarding evaluating the surgical specimens obtained by light and electron microscopy.

## Light and electron microscopic findings

The specimens obtained were evaluated by light microscopy in 1-μm plastic sections stained with toluidine blue and by transmission electron microscopic examination of ultrathin sections prepared according to standard techniques (a mixture of 2.5% glutaraldehyde and 2% formaldehyde fixation buffered in 0.1 M cacodylate followed by 2% osmium fixation in an aqueous solution).

**Fig. 4:**
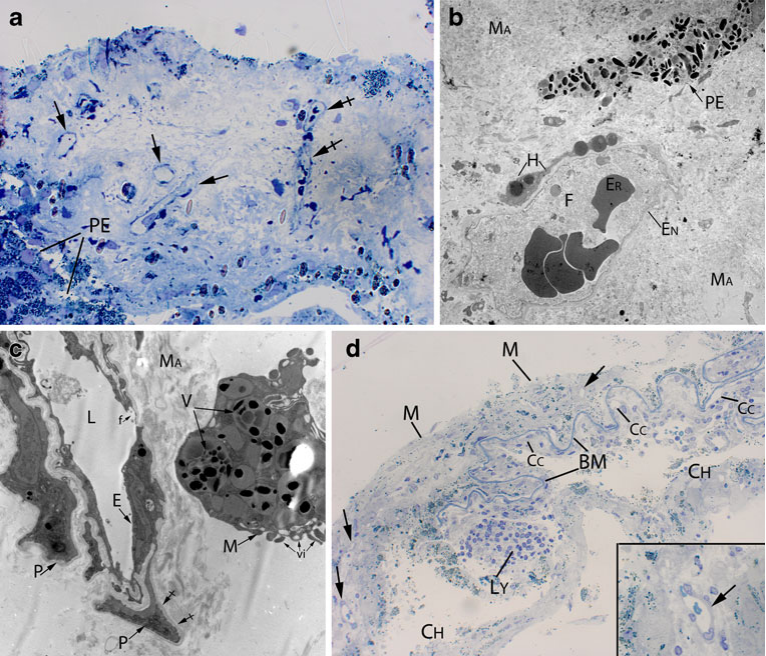
**a** Right submacular surgical specimen consists of collagen with many capillary channels (*arrows*), some of which have erythrocytes in their lumens (*crossed arrows*). Scattered pigment granules are observed, with a concentration of pigment epithelial cells (*PE*) toward the *bottom left* (Toluidine blue, ×200). **b** A nonfunctioning capillary exhibits attenuation and fragmentation of the endothelial (*EN*) cell layer in the absence of pericytes. Note the persistence of erythrocytes (*ER*) within the lumen, which is filled with microfilaments (*F*). *H* histiocyte, *PE* retinal pigment epithelial cell process, *MA* extracellular fibrocollagenous matrix (EM ×2,600). **c** A viable neovascular unit displays endothelium (*E*) with focal fenestrations (*f*) and elongated lumen (*L*). Both the endothelial cells and surrounding pericytes (*P*) are clothed by a basement membrane. The matrix (*MA*) is looser in this region and contains a macrophage (*M*) with complex phagolysosomes (*V*) and surface villi (vi). (EM ×5,800). **d** Multilaminar membrane (*M*) from the left eye has formed above Bruch’s membrane (*BM*) and contains small neovascular units (*arrows*). The adherent choriocapillaris (*Cc*) is patent. Within the choroid (*CH*) is a small collection of lymphocytes (*LY*). *Inset*: erythrocytes in a neovascular lumen (*arrow*). (Toluidine blue, ×200)

The first specimen was obtained from the right eye 20 months after the introduction of anti-VEGF therapy, 3 months after the most recent intravitreal bevacizumab injection. The light microscopic evaluation of epon sections revealed a hypocellular membrane featuring multiple widely separated capillaries in the midst of a fibrillar matrix without apparent inflammatory cells (Fig. 4a). Cellular processes of pigment-bearing cells were widely distributed throughout the tissue. Some of the vessels contained erythrocytes, which were not detected in the extracellular space. At one edge of the membrane, a cluster of pigment epithelial cells was observed.

At the ultrastructural level, no photoreceptors or other retinal elements were detected. Two types of capillaries were discovered. The predominant one contained occasional intraluminal erythrocytes but was for the most part occluded with microfibrils (Fig. 4b). The endothelial cells were attenuated and fragmented and a pericytic mantle was absent. A degenerating elongated pigment epithelial cell process was occasionally identified. The second less common type of capillary had a well-defined pericytic layer; only one such capillary was identified in the specimen (Fig. 4c). The surrounding fibrillar matrix was loose and a single macrophage was identified nearby. The pigment epithelial cell cluster exhibited a peculiar fine clumping of the nuclear chromatin and disruption of the plasmalemmas, presumably degenerative phenomena, causing the extracellular release of melanin granules.

The membranous tissue excised from the left eye 5 months after initiation of therapy and 3 months after most recent intravitreal injection comprised the inner choroid with its choriocapillaris, Bruch’s membrane, and a vascularized subretinal multilaminar cellular membrane with many capillary-type units (Fig. 4d). In the epon-embedded, light microscopic sections, no break was detected in Bruch’s membrane that demonstrated a vascular channel connecting the choriocapillaris with the subretinal neovascular units. The retinal pigment epithelial (RPE) layer was disrupted and disorganized. A prominent extracellular fibrillary matrix was not observed and photoreceptor cells were not seen. The inner choroid harbored a distinct focus of chronic inflammatory cells without follicular organization.

**Fig. 5:**
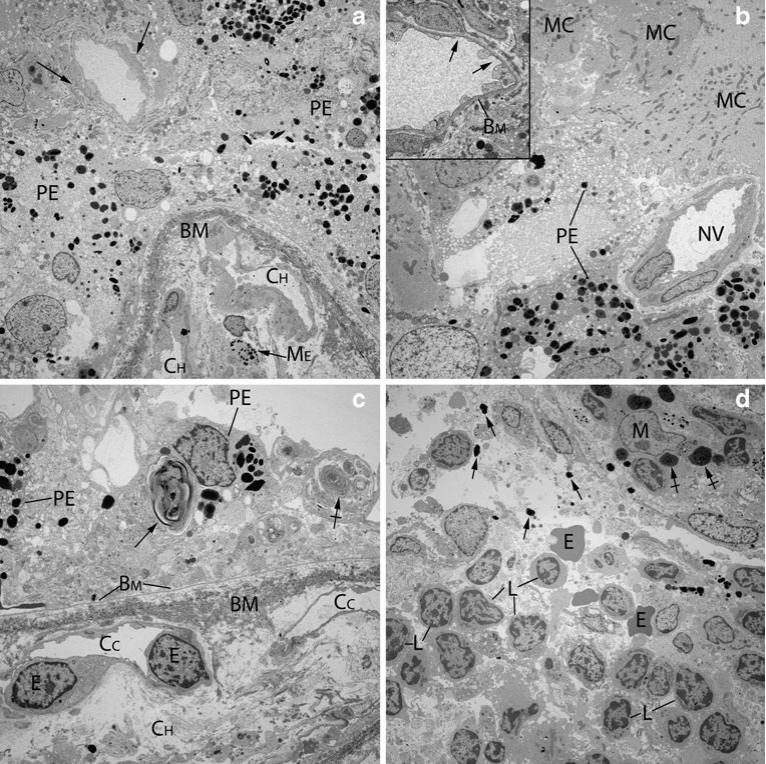
**a** In the left eye, above Bruch’s membrane (*BM*) with its elastic layer and subjacent choriocapillaris (*CH*), are two to three layers of pigment epithelial cells (*PE*) with loss of polarity. A capillary vessel (*arrows*) is present within the subretinal membrane. *ME* choroidal melanocyte (EM ×1,450). **b** Pigment epithelial cells (*PE*) approach a neovascular (*NV*) channel. The PE cells interface with broad processes of Mueller cells (*MC*) in the upper half of the field with myriad mitochondria. *Inset*: fenestrations (*arrows*) of the endothelial cells of the neoformed capillary and a thick surrounding basement membrane (*BM*) (EM ×1,950). **c** At the edge of the membrane, pigment epithelial cells (*PE*) with basement membrane (*BM*) are positioned on Bruch’s membrane (*BM*). Within the cytoplasm of the central PE cell is a degenerated compact whorled membranous body (*arrow*), another of which is also shown toward the right in the cytoplasm of an indeterminate cell (*crossed arrow*). The underlying choriocapillaris (*Cc*) with its endothelial cells (*E*) is patent. *CH* choroid (EM ×2,600). **d** An inner choroidal inflammatory infiltrate is composed of lymphocytes (*L*). Small uveal melanosomes have been released into the extracellular space (*arrows*). A large melanophage (*M*) contains complex phagolysosomes (*crossed arrows*) of engulfed uveal melanin. *E* extravasated erythrocyte (EM ×1,450)

By transmission electron microscopy, the pigment epithelium consisted of one or two layers at the center of the membrane with neovascular channels distributed among them in the absence of inflammation (Fig. 5a). One layer of RPE was present toward the periphery of the membrane. The RPE cells and neovascular channels in the middle of the lesion also interfaced with broad processes of Mueller cells without the interposition of photoreceptors or their degenerative remains (Fig. 5b). The Mueller cells were characterized by multiform mitochondria, abundant profiles of smooth surfaced endoplasmic reticulum, and the absence of pigment granules. Poorly pigmented displaced RPE cells contained rare pigment granules, profiles of rough surfaced endoplasmic reticulum and manifested segments of basement membrane material and disorganized surface villi. Extravasated erythrocytes, macrophages, lymphocytes, and conspicuous extracellular matrix material were not found in the membrane, except for the last which was limited to the pericapillary zone. The viable neoformed intramembranous capillaries displayed patent lumens with endothelial cell fenestrae and uniformly enveloping pericytes, both ensheathed by basement membranes (Fig. 5b).

At the edge of the membrane, a single layer of dyspolar pigment epithelial cells without apical villi was surrounded by whorled membranous bodies of various sizes, some of which were engulfed by the RPE cells and others by macrophages (Fig. 5c). These probably represented degenerative fragments of photoreceptor outer segments. The subadjacent choriocapillaris was patent, nondamaged and uninflamed. The focus of inner choroidal inflammatory cells was composed of mature small lymphocytes with clumped chromatin and scant cytoplasm without intermixed histiocytes and plasma cells (Fig. 5d). The choroidal melanocytes were focally damaged and had released their small pigment granules extracellularly, which had attracted melanophages (Fig. 5d).

## Discussion

This report documents for the first time the healing response following anti-VEGF treatment of CNVs and highlights possible causes of treatment failure. We also analyzed the clinical and anatomical implications of surgical removal of CNVs following treatment failure of anti-VEGF agents. There is one other report in the literature of excision of a CNV membrane following intravitreal injection of the anti-VEGF agent bevacizumab, in which only avascular fibrous tissue was described pathologically [4].

The submacular membranes removed from both eyes in our case had significantly different light and electron microscopic features. That from the right eye, which had been followed and treated over a span of 20 months prior to surgical excision (in comparison with 5 months for the left eye), was remarkably hypocellular and mainly composed of abundant fibrillocollagenous material. Apart from the presence of rare elongated processes of degenerating pigment epithelial cells or an occasional macrophage, lymphocytes and plasma cells were absent. A cluster of polygonal pigment epithelial cells at the membrane’s base near Bruch’s membrane displayed disrupted plasmalemmas and a disturbed nuclear chromatin pattern of fine clumping compatible with incipient dissolution. The ability of the RPE cells to undergo fibrous and even osseous metaplasia is well recognized [5]. The vascular endothelial cells of the membrane’s capillaries were attenuated and fragmented, and their lumens were filled with microfibrils. Pericytes were overwhelmingly absent. Only one capillary appeared functional with healthier looking constituent cells that included fenestrated endothelial cells and well-preserved pericytes. In this zone, the surrounding extracellular matrix was looser, suggesting that such vessels were of more recent origin than the others and probably related to the recurrence.

In contrast, the submacular membrane that was removed from the left eye 5 months after initiation of treatment displayed virtually no extracellular fibrillar matrix material. Instead it manifested blunted processes of Mueller cells in apposition to RPE cells. The obviously viable and intact vascular endothelial cells displayed fenestrae and were consistently surrounded by pericytes. The fenestrae support an origin of the neovascular units from the choriocapillaris, which normally possesses this feature. The growth pattern of the CNV was subretinal, in accordance with previous reports [6]. There was no evidence of a lymphohistiocytic inflammatory component accompanying the subretinal neovascularization throughout most of the membrane, as also described in a previous immunohistochemical study of CNV in PIC [7]. Olsen et al., however, reported moderate to prominent lymphocytic infiltration in four out of the six CNV specimens studied [6].

The pigment epithelial cells in the left membrane remained for the most part confined to one or two layers at the base of the membrane and were in direct apposition to broad processes of Mueller cells without the interposition of photoreceptors or their degenerative remnants. The latter were discovered at the borders of the membrane in the form of whorled lamellated membranous bodies probably representing degenerative products of outer segments. This finding suggests a primary degenerative mechanism rather than an infarction, which would have produced a more complete and broader effacement of cellular detail with a prominent inflammatory cell infiltrate. Finally, the fortuitous discovery of lymphocytes at the level of the inner choroid with sparing of the choriocapillaris lends, for the fist time, ultrastructural electron microscopic support to the hypothesis that PIC is an inflammatory disease, with the inflammation originating in the choroid. There are no other EM studies of tissue excised from a PIC case that include choroid manifesting inflammatory infiltration.

Thus, there was a striking difference in the response of the two submacular membranes to bevacizumab treatment. While different durations of the two membranes before their removal created concomitant differences in the amount of collagen deposition, the chief ultrastructural differential feature was the pronounced regression of the neoformed vessels in the right eye that lacked pericytes (in comparison with those in the left eye that possessed them). This phenomenon could be a primary feature of the neovascular units themselves or reflect a preferential secondary loss during the membrane’s evolution or treatment. The well-preserved architecture of the endothelial-lined vascular channels with a normal accompaniment of pericytes in the left eye intimates that anti-VEGF treatment had no effect on them. The finding of one capillary in the right specimen with fenestrated endothelial cells and well-preserved pericytes, while all other capillaries which were occluded manifested fragmented endothelial cells and lacked pericytes, may signify that it represented CNV nonresponsiveness to the most recent anti-VEGF treatment 3 months prior to surgery. This is further supported by the fact that in this zone the surrounding extracellular matrix was looser, suggesting more recent origin and less deposition of collagen as explained above. The capillaries with fragmented endothelial cells most likely represent the regressed CNV treated with anti-VEGF agents 20 months prior to surgery.

Pericyte-poor neovascular units have been shown to be more susceptible to anti-VEGF agents than pericyte-rich ones. We therefore hypothesize that failure to respond to anti-VEGF treatment in the left eye of our patient may be associated with the uniform presence of a pericytic component in the neovascular channels. VEGF expression in developing retinal vasculature has been observed in pericytes contacting new vessels [8]. These observations indicate that the presence of pericytes may act as a survival factor for endothelial cells and therefore increase resistance to anti-VEGF therapy. Consequently, combination therapeutic strategies might be applied to circumvent such nonresponsiveness. In this context, dual VEGF and platelet-derived growth factor-targeted therapy has been shown to be more effective in inhibiting in vivo tumor growth than either agent alone [9].

Choroidal lymphocytic infiltration was found in the left subretinal excision which included a segment of choriocapillaris and surrounding inner choroidal tissue. A small collection of lymphocytes was detected in the right eye. This finding should not be totally unexpected despite the absence of overt clinical intraocular inflammation. It supports the concept that PIC is fundamentally an inflammatory condition. None of the eyes had undergone prior surgery or photodynamic therapy that could have triggered inflammation. The presence of a focus of choroidal inflammation is also suggestive of the possibility that anti-VEGF treatment of CNVs may fail because of underlying persistent inflammation. This provides another basis for considering a combination of therapeutic modalities in addition to anti-VEGF factors to improve the efficacy of treatment in CNVs secondary to PIC. This is the first report showing persistent choroidal inflammation after anti-VEGF treatment that could possibly provoke recurrent CNVs, in contradistinction to inflammation in the membrane itself.

Available data on treatment of CNV associated to PIC are derived from small nonrandomized case series. Local and systemic treatment with steroids has not been proven effective in the treatment of CNV associated with PIC, even when administered at high doses for many months [6, 10]. Small retrospective series have suggested that PDT may be effective [10]. More recent small prospective studies of PDT in combination with either systemic steroids or 4 mg intravitreal triamcinolone of five and four eyes, respectively, showed decreased activity of CNV lesions [11, 12]. Larger lesions though did not respond. Three out of the four patients who received intravitreal triamcinolone experienced improvement of two or more lines of vision and one patient lost two or more lines at 1-year follow-up [12]. We should, however, take into account the high rate of side effects of systemic or local corticosteroid treatment.

Regarding the surgical management outcome of this case, prior anti-VEGF treatment did not appear to complicate surgical excision. In our series of three submacular surgeries in two eyes, there were no adverse events peri- or postoperatively, but the number is too small for any definite conclusions. No recurrences occurred 23 months after surgery in the right eye. CNV recurrence was noted 6 months postoperatively in the left eye. Repeat surgical excision after failed anti-VEGF treatment was again uneventful. This high rate of recurrence in our series is in accord with the results of the Submacular Surgery Trial (SST) trial in which recurrent CNV developed in 58% of surgically treated eyes by the 24-month examination [13]. Recurrences occurred in four of six eyes with CNV associated with PIC following surgical excision in the series reported by Olsen et al. [6].

Following reports from the SST trial for CNV that showed no benefit in preventing visual loss and a high rate of adverse events, submacular surgery has been virtually abandoned. SST reported on both type I membranes, which grow below the RPE and are typically secondary to AMD, and type II membranes, which grow subretinally. PIC is one of the conditions, along with an idiopathic category, myopia, and presumed ocular histoplasmosis syndrome, that are complicated by type II CNVs. The SST trial showed that surgical excision of CNV in POHS and idiopathic cases probably has a more favorable visual outcome in the subgroup of eyes with initial visual acuity 20/100 or worse [13]. A successful outcome was defined as a visual acuity better, or no more than one line worse, than at baseline. As a result, a smaller benefit of surgery, such as reduction of risk of loss of three lines of vision, which many trials have been designed to detect, could not be confirmed. Also, 25% of patients were 60 years or older; results may be more favorable in a younger cohort of patients, as large case series have suggested [14]. Still, submacular surgery carries significant risks and should be considered only in selected cases nonresponsive or nontolerant to other treatment modalities.

In conclusion, this report provides evidence that inhibition of VEGF alone cannot be relied upon to induce stable and lasting regression of subretinal neovascularization in PIC. A multimodal therapeutic approach may be necessary to disrupt multiple signaling pathways in neoangiogenesis. This is the first pathological study employing human tissue that points to pericytes as a potential critical target with the aggravating influence of inner choroidal chronic inflammation.
